# Depletion of the Adaptor Protein NCK Increases UV-Induced p53 Phosphorylation and Promotes Apoptosis

**DOI:** 10.1371/journal.pone.0076204

**Published:** 2013-09-23

**Authors:** Timothy M. Errington, Ian G. Macara

**Affiliations:** 1 Department of Microbiology, Center for Cell Signaling, University of Virginia School of Medicine, Charlottesville, Virginia, United States of America; 2 Department of Cell and Developmental Biology, Vanderbilt University Medical Center, Nashville, Tennessee, United States of America; Université de Sherbrooke, Canada

## Abstract

The cellular response to DNA damage requires the coordination of many proteins involved in diverse molecular processes. Discrete molecular pathways are becoming increasingly well understood, but the interconnectivity and coordination of multiple pathways remains less clear. We now show that NCK, an adapter protein involved in cytoskeletal responses to tyrosine kinase receptor signaling, accumulates in the nucleus in response to DNA damage and this translocation can be blocked by specific inhibition of the ATR protein kinase. Strikingly, HeLa cells depleted of NCK undergo apoptosis shortly after UV irradiation, as monitored by caspase-3 cleavage and PARP cleavage. This rapid, hyperactive apoptosis in NCK depleted cells might be p53 dependent, because loss of NCK also increased UV-induced p53 phosphorylation. Importantly, depletion of SOCS7, which is necessary for NCK nuclear translocation, phenocopies NCK depletion, indicating the nuclear accumulation of NCK is responsible for these molecular events. There are two NCK isoforms that have mostly redundant functions, and although NCK2 appears to have a greater contribution, depletion of NCK1 or NCK2, led to increased p53 phosphorylation and early apoptosis after UV exposure. These data reveal a novel function for NCK in regulating p53 phosphorylation and apoptosis, and provide evidence for interconnectedness of growth factor signaling proteins and the DNA damage response.

## Introduction

Damage to DNA can occur from endogenous sources, such as reactive oxygen species and short telomeres, or exogenous sources, such as ultraviolet light (UV) and ionizing radiation (IR) [1]. The cellular response to DNA damage involves the recognition of damage, resulting in the initiation of a signal that is transmitted to mediator and signaling kinases, which then act upon different target proteins to mount an appropriate response, such as cell cycle arrest and DNA repair, or apoptosis [2]. Failure to mount an effective DNA damage response (DDR) leads to genetic instability, with impacts on aging, development, and cancer [3]. Three key protein kinases in the DDR pathway are ATM, ATR, and DNAPK, members of the phosphoinositide-3-kinases related kinase (PIKK) family, which phosphorylate multiple proteins, including the histone variant H2AX, the checkpoint protein CHK2, and the tumor suppressor p53, to initiate a signaling cascade [4-8]. A proteomic screen for substrates of ATM and ATR revealed over 700 proteins phosphorylated in response to IR or UV, a surprising number of which are associated with signaling pathways quite distinct from the DDR, illustrating the broad potential for intersection of multiple cellular processes [9].

NCK (non-catalytic (region of) tyrosine kinase adaptor protein) is part of a family of Src homology domain containing adaptor proteins, which are composed almost entirely of protein-protein interaction domains with no known catalytic activity [10]. Similar to other members of this family, NCK has been shown to couple signals from activated receptor tyrosine kinases to downstream effectors through its various SH domains [11]. In mammals there are two isoforms of NCK, NCK1 and NCK2, which share 68% amino acid identity and have been considered functionally redundant [12-14]. NCK is predominantly cytoplasmic but, unexpectedly, continually shuttles in and out of the nucleus, as determined by the nuclear accumulation of NCK in cells treated with leptomycin B [15]. A binding partner of NCK, SOCS7 (suppressor of cytokine signaling 7) has been identified as the carrier protein that mediates the nucleo-cytoplasmic translocation of NCK [15,16]. An earlier study from our group identified an unexpected link between NCK, SOCS7, and the DDR, through the nuclear accumulation of NCK following UV damage [15].

In this study we address the functional significance of UV-induced nuclear accumulation of NCK. We discovered that depletion of NCK in HeLa cells leads to apoptosis shortly after UV damage, possibly by increased phosphorylation of p53.

## Materials and Methods

### Cell Culture, Constructs, and Transfections

HeLa, MCF7 and 293T cells were purchased from ATCC and grown in DMEM supplemented with 10% fetal calf serum. pK-GFP-NCK1, pK-GFP-NCK2, pK-myc-NCK1, and pK-myc-NCK2 constructs were generated by cloning NCK1 and NCK2 into BamHI/EcoI sites of pKGFP or pKmyc vectors. siRNAs were purchased from Thermo scientific: siControl (custom, described previously [17]), siNCK1 (M-006354-01), siNCK2 (M-0197547-01), siCHK2 (M-003256-06), siSOCS7 (M-027197-00), siNCK2#2 (GGAAGUGGCGCUCGUGCAU). Knockdown transfections with siRNA were performed with Lipofectamine RNAiMAX (Invitrogen) and transfected 16 hr after plating then transfected a second time 48 hr after the first transfection following the manufacturer suggested amount of reagent, siRNA and media. Overexpression transfections were performed with Lipofectamine 2000 (Invitrogen) as described previously [18]. Cells were treated, as indicated, 24 hr after transfections. Unless indicated otherwise, siNCK indicates equal amounts of siNCK1 and siNCK2 siRNA were transfected and GFP-NCK indicates equal amounts of GFP-NCK1 and GFP-NCK2 were transfected.

### Immunofluorescence

Cells were grown on Lab-Tek II chambers (Nunc) and, when indicated, treated with 50 J/m^2^ UV, 10 µM etoposide (Sigma) or 10 Gy IR and allowed to recover for 2 hr or 1 hr before being fixed in 3.7% paraformaldehyde in PBS. Cells were permeabilized and blocked in 0.3% saponin with 0.5% BSA in PBS for 1 hr before incubation with antibodies. Antibody incubations and washes were carried out in 0.3% saponin with 0.5% BSA in PBS. Where indicated cells were pretreated with 50 µM wortmannin (Sigma), 5 µM ATR inhibitor, VE-821 (3-amino-6-(4methylsulfonyl_phenyl-N-phenylpyrazine-2-carboxamide), (described previously [19]), (a gift from David Cortez (Vanderbilt Univ.)), or vehicle (DMSO) for 30 min prior to UV treatment and then allowed to recover 2 hr in the presence of wortmannin, ATR inhibitor, or vehicle. Primary antibodies used were: mouse anti-NCK (1:250) (BD Biosciences), rabbit anti-CHK2-pT68 (1:100) (Cell Signaling), rabbit anti-γH2AX (pS139) (1:750) (Novus Biologicals), rabbit anti-cleaved CASP3 (1:100) (Cell Signaling), mouse-anti-γH2AX (pS139) (1:250) (Millipore). Alexa Fluor-conjugated secondary antibodies (Invitrogen) were used at a dilution of 1:1,000. Nuclei were counterstained with DRAQ5 (1:500) (Cell Signaling) and mounted in Fluormount-G (Southern Biotech). Images were captured using a LSM510 Meta confocal microscope (Carl Zeiss, Thornwood, NY) using a 100x oil immersion lens (NA 1.3). Images were converted to TIFF format using ImageJ and processed using Adobe Photoshop CS4 Levels tool (Adobe Systems, Mountain View, CA) to enhance contrast. Nuclear and cytoplasmic flurorescence signal was quantified using ImageJ.

### Immunoblotting

Immunoblotting was performed as described previously [20]. When indicated cells were treated with 50 J/m^2^ UV and allowed to recover for 2 hr before being harvested. Primary antibodies used were: rabbit anti-NCK (a gift from Tony Pawson (Univ. of Toronto, Canada)), rabbit anti-cleaved CASP3 (Cell Signaling), rabbit anti-CASP3 (Cell Signaling), rabbit anti-PARP (detects total and cleaved forms) (Cell Signaling), rabbit anti-RAN (described previously [21]), rabbit anti-p53-pS15 (Cell Signaling), rabbit anti-CHK2-pT68 (Cell Signaling), sheep anti-p53 (Millipore), rabbit anti-NCK2 (a gift from Louise Larose (McGill Univ., Canada)), rabbit anti-CHK2 (Santa Cruz), rabbit anti-γH2AX (pS139) (Novus Biologicals). HRP-conjugated secondary antibodies (IgG, Jackson ImmunoResearch Laboratories; Protein-A, Millipore) were used at a dilution of 1:5,000. Band intensities were quantified using ImageJ.

### Cell Survival Assay

HeLa cells were plated and then transfected in a 96 well plate. Cells were treated with UV at 50 J/m^2^ and allowed to recover for the indicated time. Wells were assayed with CellTiter 96 AQ_ueous_ One Solution (Promega) following the manufacturer protocol.

### Reverse-Transcription (RT)-PCR

RNA was harvested with TRIzol Reagent (Invitrogen) and then treated with RQ1 RNase-free DNase (Promega) supplemented with RNasin (Promega) before a reverse transcription reaction was performed with random hexamers (Invitrogen) and SuperScript II Reverse Transcriptase (Invitrogen) supplemented with RNasin. PCR of the reverse transcribed cDNA was performed with p53, GAPDH, NCK1, NCK2, and SOCS7 primers as described previously [22-24].

### Immunoprecipitation

HeLa cells were lysed and clarified as described previously [20]. Lysates were incubated with rabbit anti-NCK or rabbit IgG antibodies overnight, rocking, at 4°C before incubation with GammaBind plus sepharose (GE Healthcare) for 4 hr. Beads were washed 3X with lysis buffer before 2X sample buffer was added and samples heated at 95°C for 5 min. All subsequent immunoblots were performed with Protein A-conjugated HRP secondary antibody to prevent detection of denatured antibody chains as described previously [25].

### Statistical analysis

Two-tailed Student’s *t*-tests were used in the statistical analysis. All graphs and statistical analysis were performed with GraphPad Prism, Version 4.0a (GraphPad Software, Inc.).

## Results

### NCK Accumulates in the Nucleus Following DNA Damage

**Figure 1:**
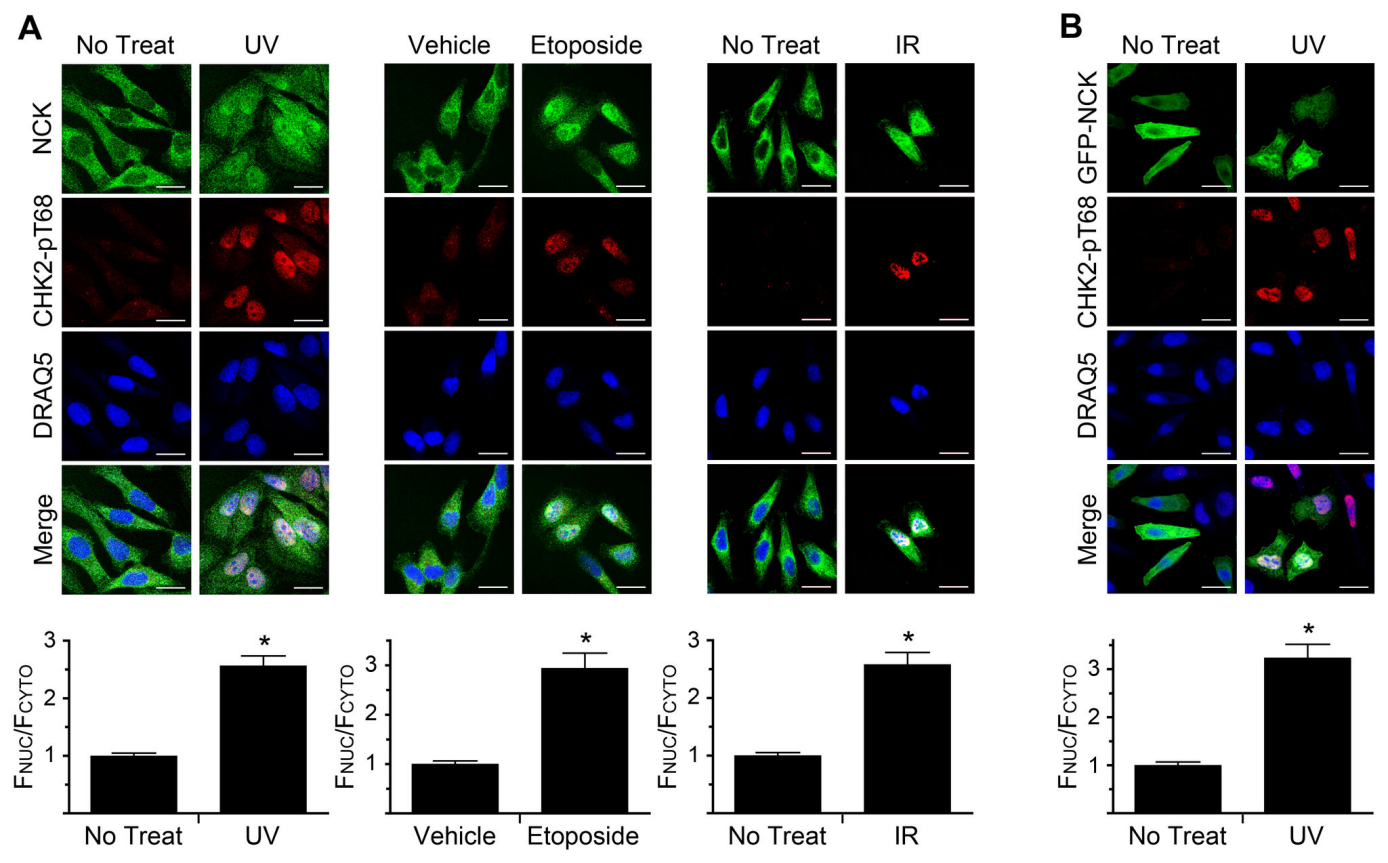
DNA damage induces the nuclear accumulation of NCK. (A) HeLa cells were treated with 50 J/m^2^ UV, 10 µM Etoposide, or 10 Gy IR and allowed to recover for 2 hr, 1 hr, and 1 hr, respectively, before being fixed and stained with the indicated antibodies and DRAQ5 to visualize nuclei. Scale bars are 20 µm. All images are confocal sections. (B) HeLa cells transfected with GFP-NCK1 and GFP-NCK2 were treated with 50 J/m^2^ UV and allowed to recover for 2 hr before being fixed and stained with indicated antibodies and DRAQ5. Bar graphs below each panel are ratio of nuclear to cytoplasmic fluorescence of endogenous NCK, A, or GFP-NCK, B, with no treat, or vehicle, defined as 1. n = 19-82; error bars represent SE; (*) P < 0.0001.

Previously, we had discovered that NCK, which constitutively shuttles between the cytoplasm and nucleus and is primarily cytoplasmic in undamaged cells, accumulates in the nucleus in response to UV irradiation [15]. We sought to confirm this observation and expand it to other types of DNA damage. HeLa cells were irradiated with UV or IR, or treated with the topoisomerase inhibitor etoposide, and then stained to determine NCK localization with a specific NCK antibody (Figure S1A). Phosphorylated CHK2 (pT68) was used to monitor whether the treatments had triggered an appropriate DDR [26]. In all cases, NCK accumulated in the nuclei of damaged cells, versus a primarily cytoplasmic staining in untreated, or vehicle-treated, cells (Figure 1A). This result is further supported by a similar relocalization of ectopically expressed GFP-NCK after UV treatment (Figure 1B). Moreover, similar results were obtained using MCF7 cells, with NCK accumulating in the nucleus following UV treatment (Figure S1B). These data show that nuclear relocalization of NCK occurs in response to multiple types of DNA damage, occurs in different cell types, and correlates with CHK2 phosphorylation. Therefore, we focused on HeLa cells and UV irradiation to further study the significance of nuclear accumulation of NCK in response to DNA damage.

### NCK Nuclear Accumulation Requires the Activity of the PIKK Family Member ATR

We next sought to determine the upstream signaling requirements for NCK nuclear accumulation. An early event in response to DNA damage is the activation of the PIKK family of protein kinases, which phosphorylate multiple downstream targets including histone H2AX, p53, and the checkpoint kinase, CHK2 [8]. Because CHK2 phosphorylation correlates with NCK nuclear accumulation after UV irradiation, we first tested the requirement for CHK2 in this process by RNAi. However, despite efficient knockdown of the kinase, NCK still accumulated in the nucleus following UV irradiation, indicating that CHK2 is not required for NCK relocalization and that NCK acts upstream, or parallel, to the checkpoint kinase (Figure S2A,B). Phosphorylation of the histone variant H2AX on serine 139 (γH2AX) was, as expected, also unaffected by depletion of CHK2 (Figure S2B,C).

**Figure 2:**
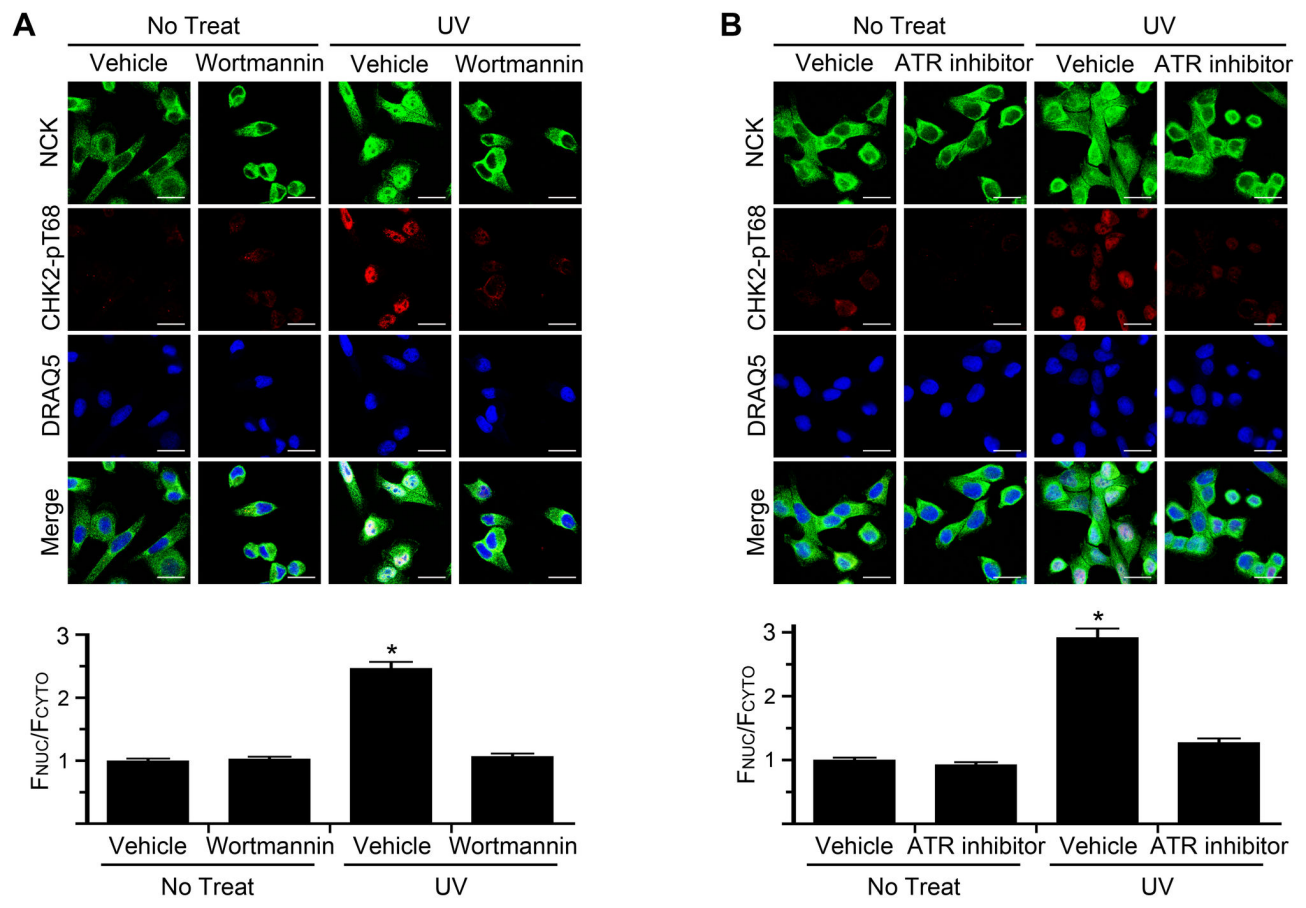
ATR activity is necessary for nuclear accumulation of NCK. (A) HeLa cells were pretreated for 30 min with 50 µM wortmannin and then treated with 50 J/m^2^ UV and allowed to recover for 2 hr in the presence of wortmannin before being fixed and stained with the indicated antibodies and DRAQ5. Scale bars are 20 µm. All images are confocal sections. Bar graph below is ratio of nuclear to cytoplasmic fluorescence of NCK with vehicle pretreated, no treat, defined as 1. n = 125-174; error bars represent SE; (*) P < 0.0001. (B) Same as in A, except 5 µM ATR inhibitor (VE-821) was used, and n = 79-117.

We next asked if NCK nuclear accumulation is dependent on PIKK kinase activity. To first test the general requirement for these kinases in UV-induced NCK nuclear accumulation we used the PIKK inhibitor wortmannin [27]. Treatment of cells with wortmannin did not affect the untreated cytoplasmic localization of NCK, but did prevent the UV-induced nuclear accumulation of NCK, compared to vehicle treatment (Figure 2A). This result indicates that NCK nuclear accumulation is likely dependent on the activity of the PIKK family. To further elucidate which PIKK family member was responsible for UV-induced NCK nuclear translocation, specific inhibitors were tested. Treatment of cells with a potent and specific ATR kinase inhibitor, VE-821 [19], prevented the nuclear accumulation of NCK compared to vehicle treatment (Figure 2B). This result indicates that NCK nuclear accumulation in response to UV damage is dependent on ATR activity.

### Depletion of NCK Results in the Hyper-activation of DNA Damage-induced Apoptosis

**Figure 3:**
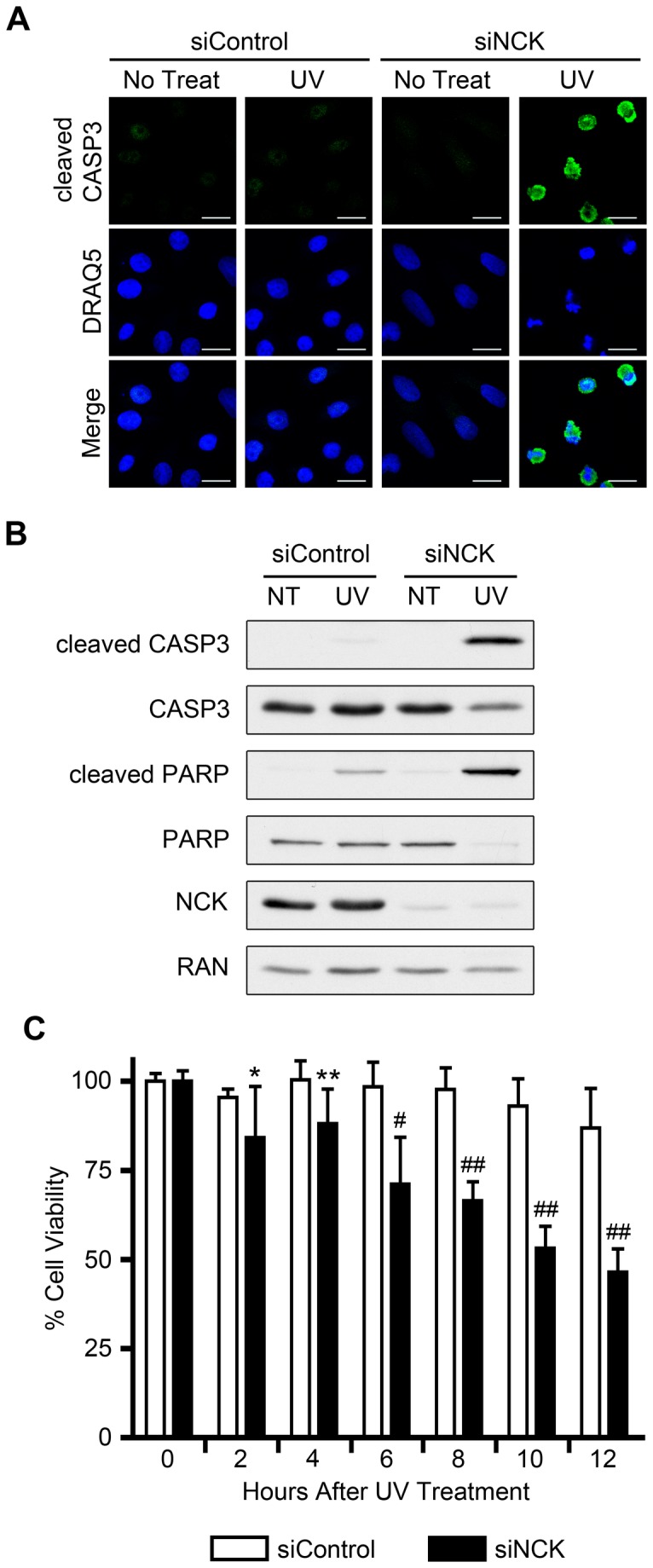
Loss of NCK causes early UV-induced apoptosis in HeLa cells. (A) HeLa cells transfected with control or NCK1 and NCK2 siRNA were treated with 50 J/m^2^ UV and allowed to recover for 2 hr before being fixed and stained with the indicated antibody and DRAQ5. Scale bars are 20 µm. All images are confocal sections. section n = 3. (B) Cells were treated as in A, and equal amounts of lysates were immunoblotted for cleaved CASP3, total CASP3, cleaved PARP, total PARP, and NCK. RAN was used as a loading control. n = 4. (C) HeLa cells transfected with control or NCK1 and NCK2 siRNA were treated with 50 J/m^2^ UV and allowed to recover for the indicated amount of time before cell viability was assayed. Percent cell viability for each siRNA at time 0 hr (no treat), is defined as 100%. n = 8; error bars represent SE; (*) P < 0.05, (**) P < 0.01, (#) P < 0.001, (# #) P < 0.0001.

We next examined if NCK is required for the cellular response to DNA damage. Remarkably, when HeLa cells were depleted of NCK (both isoforms) and irradiated with UV, cell death appeared to occur within 2 hrs after treatment, as opposed to UV-irradiated control cells, all of which showed no change in morphology over this period (Figure S3). There are various forms of cell death, but a hallmark of cells undergoing apoptosis is the induction of the caspase cascade, leading to cleavage, and thus activation of downstream caspases, specifically caspase-3 (cleaved CASP3) [28]. Strikingly, cells depleted of NCK were positive for cleaved caspase-3 staining within 2 hr after UV treatment (Figure 3A). As determined by immunoblotting, both cleaved CASP-3 and cleaved PARP, a downstream target of the caspases, were increased in UV-irradiated NCK-depleted cells compared to control (Figure 3B). To further examine the effects of NCK-depletion on apoptosis, cell viability was monitored following UV irradiation. While control cells showed a modest decrease in cell viability 12 hr after UV irradiation, depletion of NCK resulted in a significant decrease in cell viability as early as 2 hr after UV treatment that further decreased to less than 50% after 12 hr (Figure 3C). These data show that loss of NCK results in unusually rapid UV-induced apoptosis.

### Depletion of SOCS7 Blocks UV-Induced Nuclear Accumulation of NCK, and Hyper-Activates DNA Damage-Induced Apoptosis

**Figure 4:**
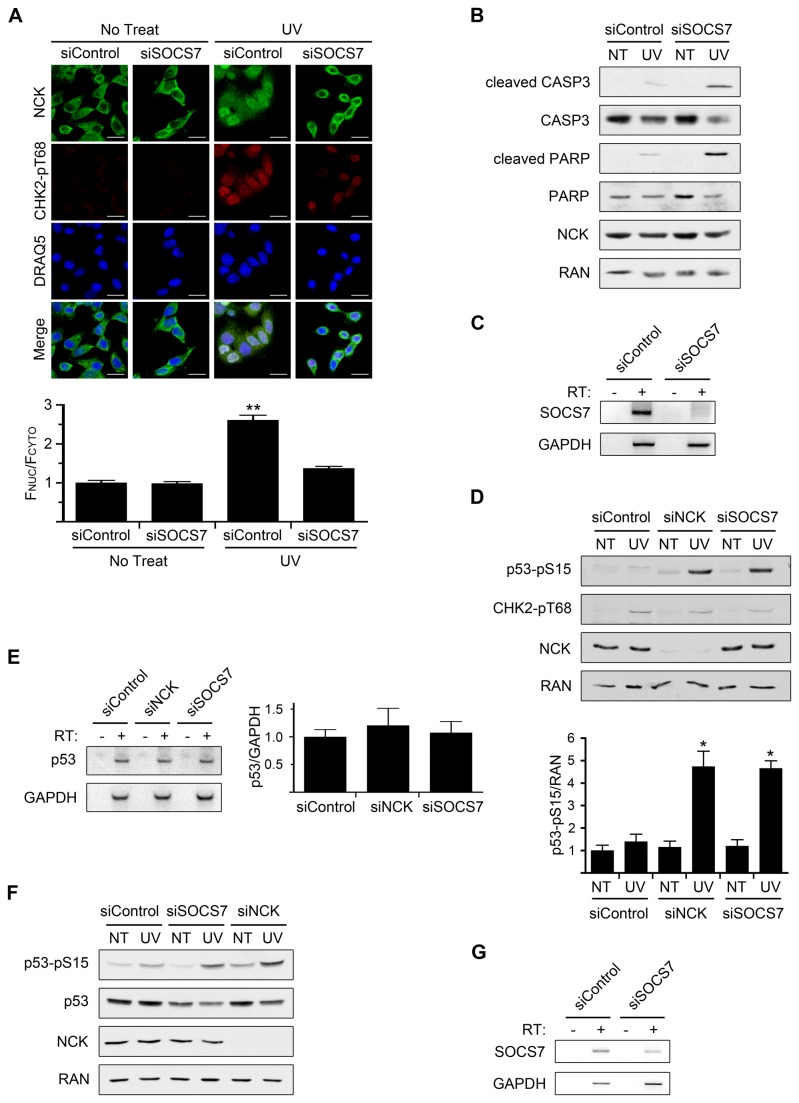
Loss of SOCS7 prevents nuclear accumulation of NCK and results in early UV-induced apoptosis and elevated p53 phosphorylation. (A) HeLa cells transfected with control or SOCS7 siRNA were treated with 50 J/m^2^ UV and allowed to recover for 1 hr before being fixed and stained with the indicated antibodies and DRAQ5. Scale bars are 20 µm. All images are confocal sections. Bar graphs below are ratio of nuclear to cytoplasmic fluorescence of NCK with siControl, no treat, defined as 1. n = 43-67; error bars represent SE; (**) P < 0.0001. (B) HeLa cells were treated as in A, and equal amounts of lysates were immunoblotted for cleaved CASP3, total CASP3, cleaved PARP, total PARP, and NCK. RAN was used as a loading control. n = 3. (C) RT-PCR of SOCS7 mRNA from HeLa cells transfected with control or SOCS7 siRNA. GAPDH was used as an internal control. RT = reverse transcriptase. n = 3. (D) HeLa cells transfected with control, NCK1 and NCK2, or SOCS7 siRNA were treated with 50 J/m^2^ UV and allowed to recover for 2 hr before lysates were prepared. Equal amounts of lysates were immunoblotted for p53-pS15 (phospho-specific), CHK2-pT68 (phospho-specific), and NCK. RAN was used as a loading control. Band intensities were measured with ImageJ. Bar graph is ratio of p53-pS15 band intensity to RAN band intensity with siControl, no treat (NT), defined as 1. n = 4; error bars represent SE; (*) P < 0.005. (E) RT-PCR of p53 mRNA from HeLa cells transfected with control, NCK1 and NCK2, or SOCS7 siRNA. GAPDH was used as an internal control. Bar graph is ratio of p53 mRNA to GAPDH mRNA with siControl ratio defined as 1. n = 4; error bars represent SE. (F) 293T cells transfected with control, NCK1 and NCK2, or SOCS7 siRNA were treated with 50 J/m^2^ UV and allowed to recover for 2 hr before lysates were prepared. Equal amounts of lysates were immunoblotted for p53-pS15, p53 (total protein), and NCK. RAN was used as a loading control. n = 3. (G) RT-PCR of SOCS7 mRNA from 293T cells transfected with control or SOCS7 siRNA. GAPDH was used as an internal control. n = 3.

Previously, we had discovered that SOCS7 is necessary for the nuclear translocation of NCK [15]. Thus, through depletion of SOCS7, we could test specifically the requirement for nuclear accumulation of NCK during DNA damage. We first sought to confirm that SOCS7 is necessary for NCK translocation in response to UV irradiation. Depletion of SOCS7 from HeLa cells did not affect the untreated cytoplasmic localization of NCK, but did prevent the nuclear accumulation of NCK, compared to control, following UV (Figure 4A). This result confirms our previous data that NCK nuclear accumulation is dependent on SOCS7. Importantly, however, loss of SOCS7 also resulted in early UV-induced apoptosis as assessed by immunoblots of lysates from SOCS7 depleted cells, which showed increased cleaved CASP3 and cleaved PARP compared to control cells (Figure 4B). Because of the low expression of SOCS7 at the protein level and the lack of a suitable antibody, we were unable to detect SOCS7 by immunoblot, but confirmed silencing at the mRNA level (Figure 4C). These data show that blocking NCK nuclear accumulation, through the knockdown of SOCS7, phenocopies NCK depletion, causing rapid UV-induced apoptosis.

### UV-induced p53 Phosphorylation is Elevated in NCK, or SOCS7, Depleted Cells

In our previous study, mouse embryonic fibroblasts cell lines null for NCK1 and NCK2 were used to examine the effect of NCK on p53 phosphorylation following DNA damage. These cells were unable to induce PIKK-dependent phosphorylation of p53 on serine 18 (mouse homolog to human serine 15) following UV irradiation [15]. In contrast, however, HeLa cells depleted of either NCK, or SOCS7, exhibited a dramatic increase in p53 phosphorylation on serine 15 after UV treatment, as compared to control cells (Fig. 4D). We asked if this increase was due to an increase in p53 transcript levels in NCK, or SOCS7, depleted cells, but as determined by RT-PCR, there was no difference with control cells (Fig. 4E). Since HeLa cells have a low level of p53 protein due to the human papillomavirus E6 oncoprotein, we tested another cell line to confirm this result [29]. Using 293T cells we observed the same UV-induced increase in p53 phosphorylation in NCK, or SOCS7, depleted cells compared to control cells (Figure 4F,G). However, there was no effect on total p53 levels, indicating that the increased phosphorylation was not due to increased protein levels (Figure 4F). Taken together, these data strongly suggest that elevated UV-induced p53 phosphorylation is correlated with a loss of nuclear NCK.

### Contributions of NCK Isoforms on UV-Induced p53 Phosphorylation and Apoptosis

**Figure 5:**
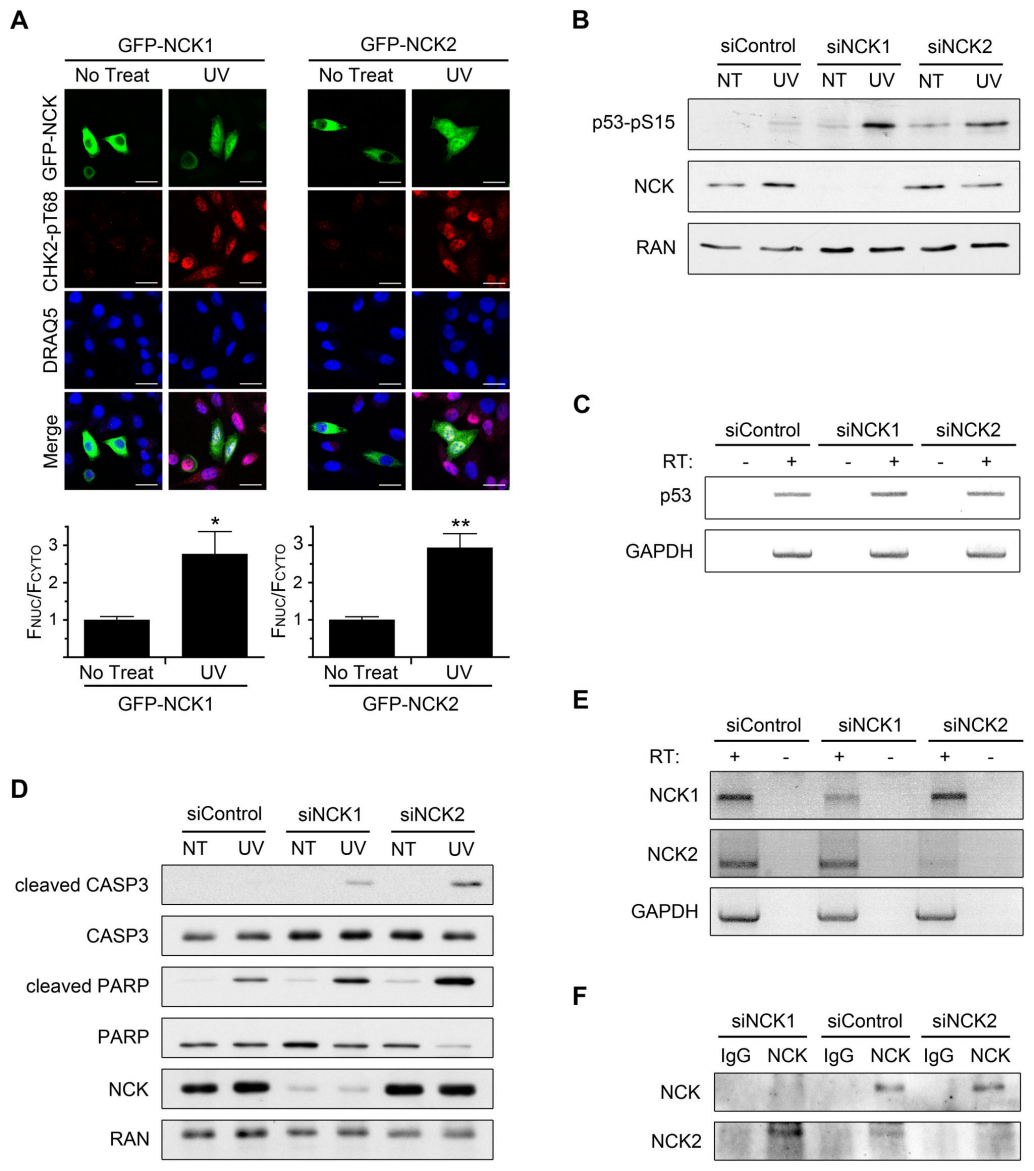
Specific contributions of the NCK isoforms. (A) HeLa cells transfected with GFP-NCK1 or GFP-NCK2 were treated with 50 J/m^2^ UV and allowed to recover for 2 hr before being fixed and stained with indicated antibodies and DRAQ5. Scale bars are 20 µm. All images are confocal sections. Bar graphs below each panel are ratio of nuclear to cytoplasmic fluorescence of GFP-NCK with no treat defined as 1. n = 14-28; error bars represent SE; (*) P < 0.001, (**) P < 0.0001. (B) HeLa cells transfected with control, NCK1, or NCK2 siRNA were treated with 50 J/m^2^ UV and allowed to recover for 2 hr before lysates were prepared. Equal amounts of lysates were immunoblotted for p53-pS15 (phospho-specific) and NCK. RAN was used as a loading control. n = 3. (C) RT-PCR of p53 mRNA from HeLa cells transfected with control, NCK1, or NCK2 siRNA. GAPDH was used as an internal control. RT = reverse transcriptase. n = 4. (D) HeLa cells transfected with control, NCK1, or NCK2 siRNA were treated with 50 J/m^2^ UV and allowed to recover for 2 hr before lysates were prepared. Equal amounts of lysates were immunoblotted for cleaved CASP3, total CASP3, cleaved PARP, total PARP, and NCK. RAN was used as a loading control. n = 3. (E) RT-PCR of NCK1 and NCK2 mRNA from HeLa cells transfected with control, NCK1, or NCK2 siRNA. GAPDH was used as an internal control. n = 3. (F) Cell lysates from HeLa cells transfected with control, NCK1, or NCK2 siRNA were immunoprecipitated with a NCK antibody, which detects NCK 1 and NCK2, or IgG control, and immunoblotted for NCK or NCK2. n = 4.

Two isoforms of NCK exist and are expressed in human cells [30]. However, the antibodies used in the above experiments are not selective for either isoform (Fig. S4A), and knockdowns were performed with siRNAs against both transcripts. Therefore, we next asked if the UV damage response phenotype might be specific to one or the other isoform. First, HeLa cells transfected with either GFP-NCK1 or GFP-NCK2 showed mostly cytoplasmic fluorescence, and nuclear accumulation occurred to similar extents for both constructs following UV irradiation (Fig. 5A). The loss of each NCK isoform was then tested to determine the effect on p53 phosphorylation. Increased UV-induced p53 phosphorylation was observed after knockdown of either isoform (Fig. 5B), an effect that was not due to any change of the p53 transcript (Fig. 5C).

We next asked if there is a differential effect of silencing NCK1 versus NCK2 on apoptosis. While the depletion of either isoform resulted in an increase in cleaved CASP3 and cleaved PARP compared to control, the loss of the NCK2 isoform caused a greater increase compared to NCK1-depleted cells (Fig. 5D). This result is particularly striking because NCK2 is expressed at a much lower level than NCK1 in these cells. We base this conclusion on the observation that although the NCK antibody used in the immunoblots detects both NCK isoforms (Fig. S4A), depletion of NCK2 did not cause a significant decrease of the total NCK detected (Fig. 5B,D). We confirmed the knockdown of each isoform at the mRNA level with isoform-specific primers (Fig. 5E). Using a NCK2-specific antibody (Figure S4A) we were also able to detect the loss of NCK2 at the protein level, although only after immunoprecipitation (IP) of total NCK from cells (Figure 5F). NCK2-specific silencing reduced the level of NCK2 detected in the pan-NCK IP. As would be expected, NCK1-specific silencing decreased the amount of total NCK in the IP; interestingly, however, NCK1 depletion increased the amount of immunoprecipitated NCK2 compared to control, probably because of reduced competition for binding to the pan-NCK antibody (Figure 5F).

Finally, we also further confirmed the effect of NCK2 depletion on UV-induced p53 phosphorylation and apoptosis by performing the experiments with another NCK2 specific siRNA (Figure S4B-E). Taken together, these data indicate both NCK isoforms undergo UV-induced nuclear accumulation, their loss is correlated with an increase in p53 phosphorylation, and they contribute to post-UV viability, although it appears that loss of NCK2 might have a more prominent role in the apoptotic response.

## Discussion

The cellular response to DNA damage involves a cascade of signaling events initiated by the members of the PIKK family [2]. This network of phosphorylation events has been demonstrated to encompass proteins involved in multiple cellular pathways, but little is understood about which proteins are involved and how they intersect with the core DNA signaling pathway [9]. The adaptor protein NCK, and SOCS7, a binding partner required for its nuclear-cytoplasmic shuttling, were previously shown to accumulate in the nucleus following UV damage [15]. We found that the nuclear accumulation of NCK occurs in response to different types of DNA damage and requires PIKK signaling. Based on the use of a specific ATR inhibitor, the nuclear accumulation of NCK in response to UV irradiation appears to be dependent on ATR kinase activity, consistent with previous studies identifying ATR as the primary PIKK family member activated in response to UV damage [31].

Cells exposed to UV damage can survive through DNA repair; however, if damage is too severe, or there are defects in repair or other protective mechanisms, cell death usually occurs via p53-dependent apoptosis [32]. In our experiments we used a UV dose shown to induce apoptosis, but not until 12-16 hr after exposure to UV [33,34]. Remarkably, in NCK-depleted cells, this apoptosis event started to occur within 2 hr, indicating that NCK plays a key anti-apoptotic role following UV treatment. Importantly, depletion of SOCS7, which blocks the UV-induced nuclear accumulation of NCK, also induced rapid apoptosis. This result suggests that the anti-apoptotic role of NCK is dependent on its nuclear translocation during the DDR. However, it is possible nuclear accumulation of SOCS7 during the DDR could also contribute to this result if there is impaired UV-induced SOCS7 nuclear translocation in NCK depleted cells.

Our data suggest that the early apoptotic events that occur in NCK, or SOCS7, depleted cells might be mediated by p53. Phosphorylation of p53 on serine 15 occurs rapidly in response to DNA damage and has been proposed as a priming event for other modifications to occur [5,35]. Once activated, p53 can cause cell cycle arrest or induce cells to undergo apoptosis [32,36]. Since NCK does not possess intrinsic enzyme activity, it ability to suppress p53 phosphorylation and apoptosis is likely mediated through an associated protein, perhaps recruiting a phosphatase to p53, or activating a negative regulator of p53 S15 phosphorylation. Although numerous proteins bind NCK, interaction studies have not yet been investigated in the context of DNA damage [37].

In our earlier work, mouse embryonic fibroblasts (MEFs) null for NCK showed a decrease in UV-induced p53 phosphorylation compared to wild-type cells [15], in contrast to the effects described here for HeLa and 293T cells depleted of NCK (Figure 4D,F). However, these MEFs were cell lines that might have undergone additional genetic changes during culture, or in response to long-term loss of NCK. With this in mind, we attempted to rescue the NCK knockout mouse embryonic fibroblasts with human NCK, but despite expression of the proteins, the defect in p53 phosphorylation was not reversed (data not shown). Moreover, we found that siRNA mediated knockdown of NCK, or SOCS7, in wild-type primary mouse embryonic fibroblasts also increased p53 phosphorylation compared to control siRNA, similar to the observations reported in this study (data not shown). We conclude, therefore, that the lack of response to UV irradiation in the NCK knockout MEFs was an artifact of the MEF cell lines, and that for multiple mouse and human cell types the loss of NCK causes an increase in p53 phosphorylation in response to DNA damage.

The two NCK isoforms are considered to be functionally redundant, because the knockout of either isoform in mice has no detectable effect, but the double knockout mouse is embryonic lethal [14]. In our study, we sought to determine if the NCK isoforms are functionally redundant in their response to DNA damage. Both NCK isoforms translocate to the nucleus following UV irradiation and their loss correlates with an increase in p53 phosphorylation, and results in early apoptosis. This suggests the two isoforms are able to compensate for each other. However, NCK2 might have a more prominent role in the apoptotic response since it displayed greater caspase activation when depleted compared to the loss of NCK1, despite being expressed at lower levels. Important questions for the future concern whether there are different binding partners of NCK1 and NCK2 following DNA damage, whether the UV-induced increase in p53 phosphorylation and increase in apoptosis are causative or correlative, and whether NCK is involved in other types of cellular stress or if it is specific to DNA damage.

## Supporting Information

Figure S1 **NCK antibody is specific and UV-induced nuclear accumulation of NCK occurs in other cell lines.** (A) HeLa cells transfected with control or NCK1 and NCK2 siRNA were fixed and stained with the indicated antibody and DRAQ5. Scale bars are 20 µm. All images are confocal section. n = 4. (B) MCF7 cells were treated with 50 J/m^2^ UV and allowed to recover for 2 hr before being fixed and stained with indicated antibodies and DRAQ5. Scale bars are 20 µm. All images are confocal sections. Bar graph below is ratio of nuclear to cytoplasmic fluorescence of NCK with no treat defined as 1. n = 20-27; error bars represent SE; (*) P < 0.0001.
(TIF)

Figure S2 **Loss of CHK2 does not alter nuclear accumulation of NCK.** (A) HeLa cells transfected with control or CHK2 siRNA were treated with 50 J/m^2^ UV and allowed to recover for 2 hr before being fixed and stained with the indicated antibodies and DRAQ5. Scale bars are 20 µm. All images are confocal sections. Bar graph below is ratio of nuclear to cytoplasmic fluorescence of NCK with siControl, no treat, defined as 1. n = 8-73; error bars represent SE. (B) Cells were treated as in A, and equal amounts of lysates were immunoblotted for CHK2 and γH2AX. RAN was used as a loading control. * indicates non-specific band. n =3 (C) HeLa cells transfected with control or CHK2 siRNA were treated with 50 J/m^2^ UV and allowed to recover for 2 hr before being fixed and stained with the indicated antibodies. Scale bars are 20 µm. All images are confocal section n = 3.
(TIF)

Figure S3 **Loss of NCK causes early UV-induced cell death in HeLa cells.** HeLa cells transfected with control or NCK1 and NCK2 siRNA were treated with 50 J/m^2^ UV and allowed to recover for 2 hr before being fixed and stained with the indicated antibodies and DRAQ5. Scale bars are 20 µm. All images are confocal section n = 3.
(TIF)

Figure S4 **Isoform specificity of NCK antibodies used and experiments with additional NCK2 siRNA.** (A) Equal amounts of lysates from 293T cells transfected with myc-vector, myc-NCK1, or myc-NCK2 were immunoblotted with indicated NCK antibodies and Myc. IB indicates antibody was used for immunoblots. IP indicates antibody was used for immunoprecipitations. IF indicates antibody was used for immunofluorescence. ^ indicates endogenous NCK. * indicates non-specific band. n = 3. (B) HeLa cells transfected with control, or NCK2 siRNA#2 were treated with 50 J/m^2^ UV and allowed to recover for 2 hr before lysates were prepared. Equal amounts of lysates were immunoblotted for p53-pS15 (phospho-specific). RAN was used as a loading control. n = 3. (C) Cells were treated the same as in B, and equal amounts of lysates were immunoblotted for cleaved CASP3, total CASP3, cleaved PARP, and total PARP. RAN was used as a loading control. n = 3. (D) RT-PCR of NCK2 mRNA from HeLa cells transfected with control, or NCK2 siRNA#2. GAPDH was used as an internal control. RT = reverse transcriptase. n = 3. (E) Cell lysates from HeLa cells transfected with control, or NCK2 siRNA#2 were immunoprecipitated with a NCK antibody, which detects NCK 1 and NCK2, or IgG control, and immunoblotted for NCK or NCK2. n = 3.
(TIF)
