# Supplementary figures and images for: Depletion of the Adaptor Protein NCK Increases UV-Induced p53 Phosphorylation and Promotes Apoptosis
Source: PLoS One. 2013 Sep 23;8(9):e76204. doi: 10.1371/journal.pone.0076204 (PMC3781058; doi:10.1371/journal.pone.0076204)

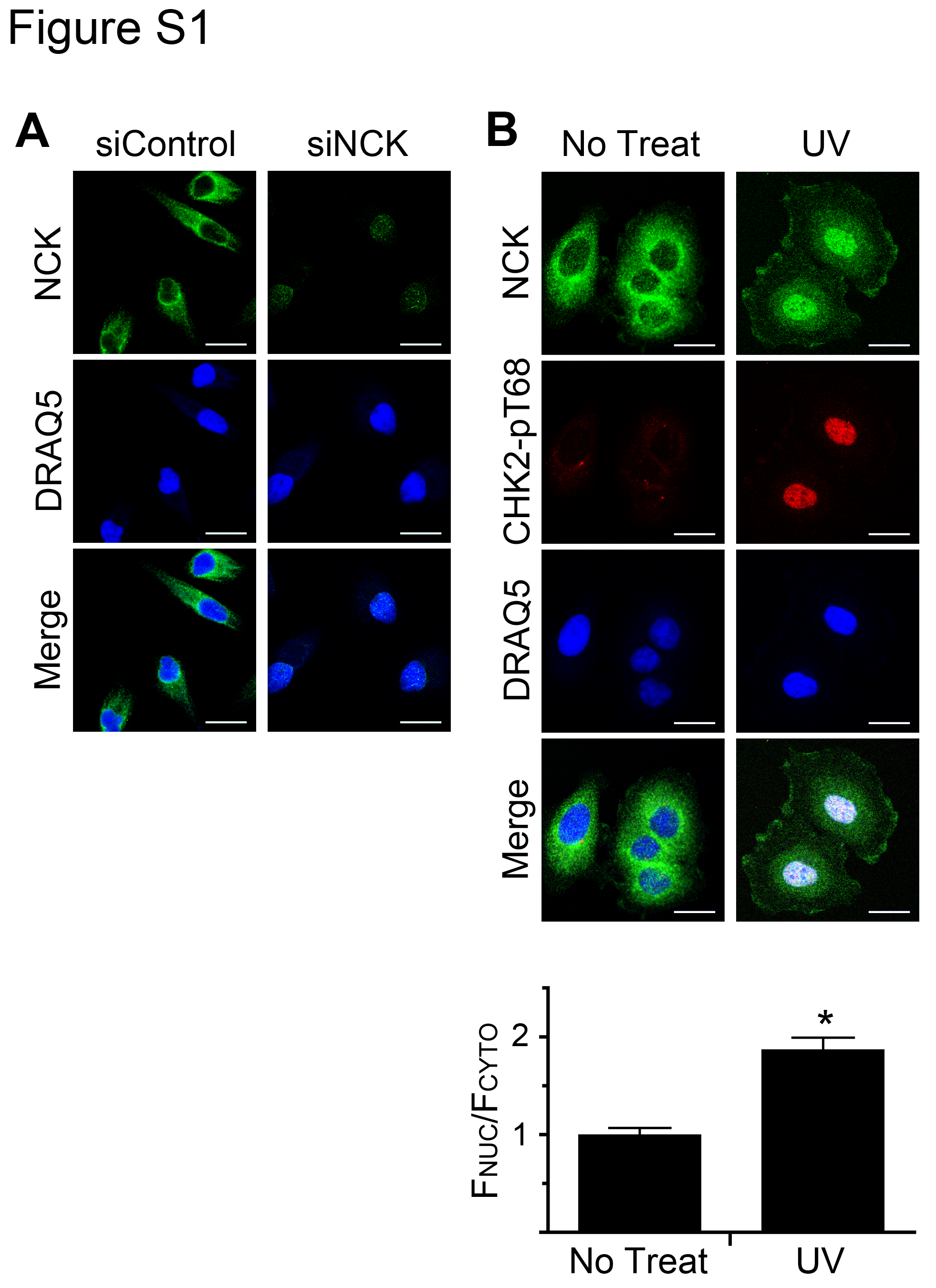

Supplement: Figure S1 — NCK antibody is specific and UV-induced nuclear accumulation of NCK occurs in other cell lines. (A) HeLa cells transfected with control or NCK1 and NCK2 siRNA were fixed and stained with the indicated antibody and DRAQ5. Scale bars are 20 µm. All images are confocal section. n = 4. (B) MCF7 cells were treated with 50 J/m2 UV and allowed to recover for 2 hr before being fixed and stained with indicated antibodies and DRAQ5. Scale bars are 20 µm. All images are confocal sections. Bar graph below is ratio of nuclear to cytoplasmic fluorescence of NCK with no treat defined as 1. n = 20-27; error bars represent SE; (*) P < 0.0001. (TIF) [file pone.0076204.s001.tif]

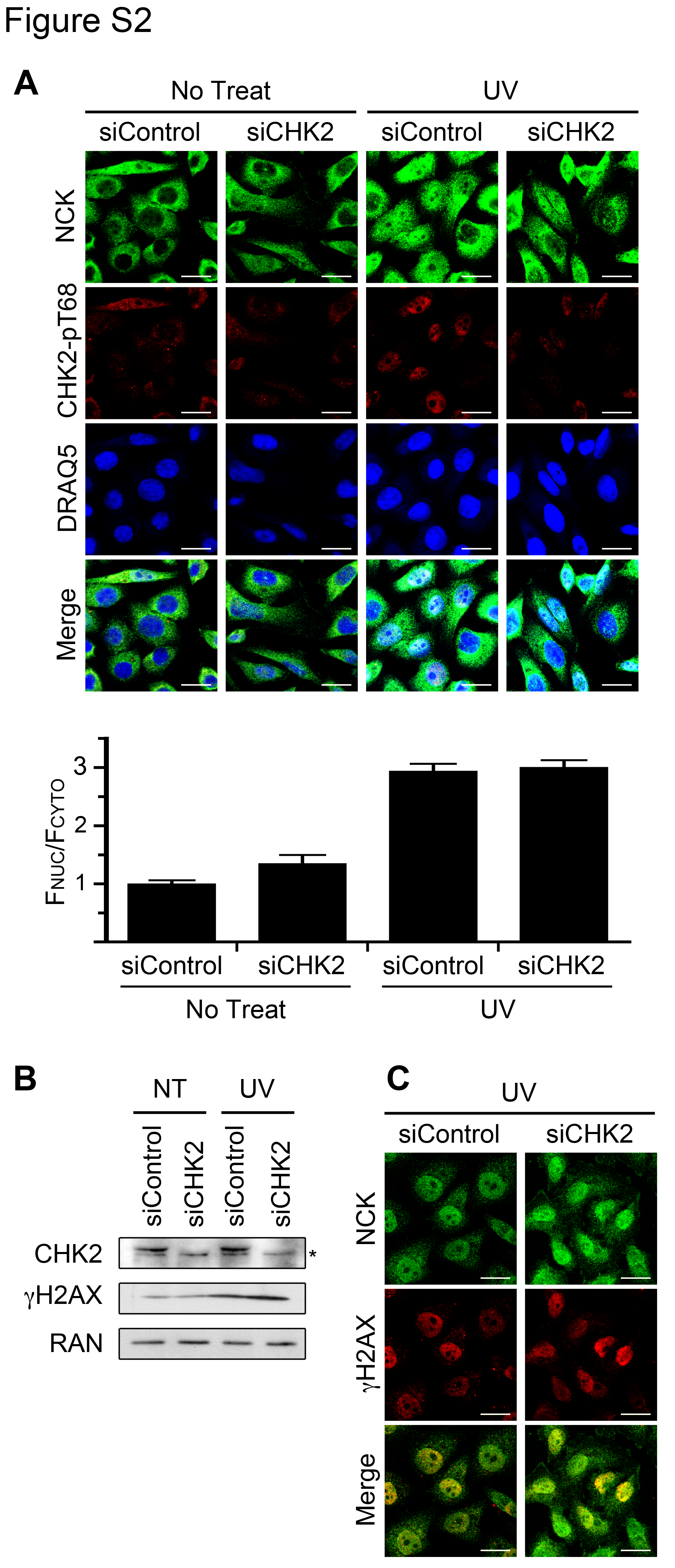

Supplement: Figure S2 — Loss of CHK2 does not alter nuclear accumulation of NCK. (A) HeLa cells transfected with control or CHK2 siRNA were treated with 50 J/m2 UV and allowed to recover for 2 hr before being fixed and stained with the indicated antibodies and DRAQ5. Scale bars are 20 µm. All images are confocal sections. Bar graph below is ratio of nuclear to cytoplasmic fluorescence of NCK with siControl, no treat, defined as 1. n = 8-73; error bars represent SE. (B) Cells were treated as in A, and equal amounts of lysates were immunoblotted for CHK2 and γH2AX. RAN was used as a loading control. * indicates non-specific band. n =3 (C) HeLa cells transfected with control or CHK2 siRNA were treated with 50 J/m2 UV and allowed to recover for 2 hr before being fixed and stained with the indicated antibodies. Scale bars are 20 µm. All images are confocal section n = 3. (TIF) [file pone.0076204.s002.tif]

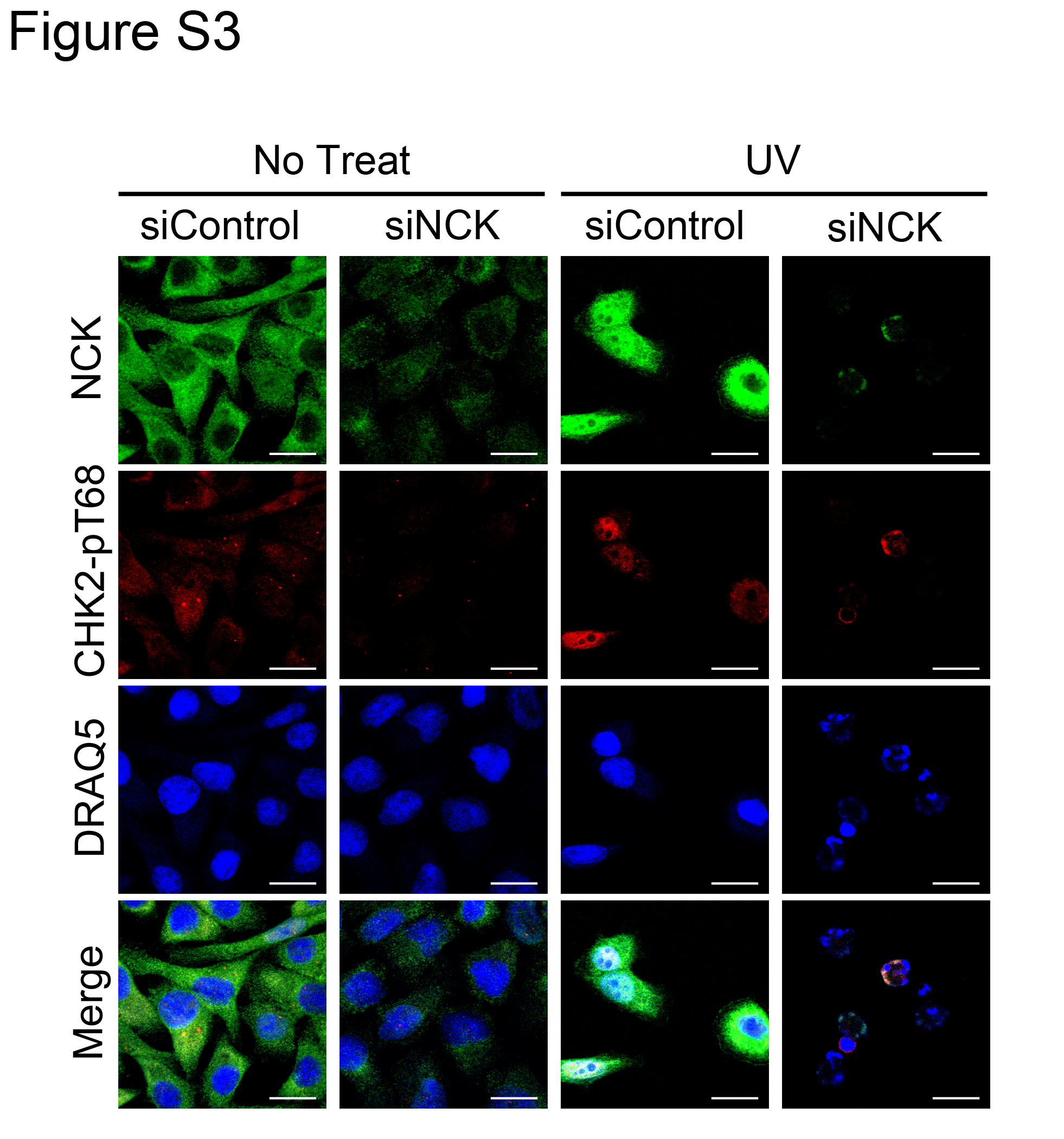

Supplement: Figure S3 — Loss of NCK causes early UV-induced cell death in HeLa cells. HeLa cells transfected with control or NCK1 and NCK2 siRNA were treated with 50 J/m2 UV and allowed to recover for 2 hr before being fixed and stained with the indicated antibodies and DRAQ5. Scale bars are 20 µm. All images are confocal section n = 3. (TIF) [file pone.0076204.s003.tif]

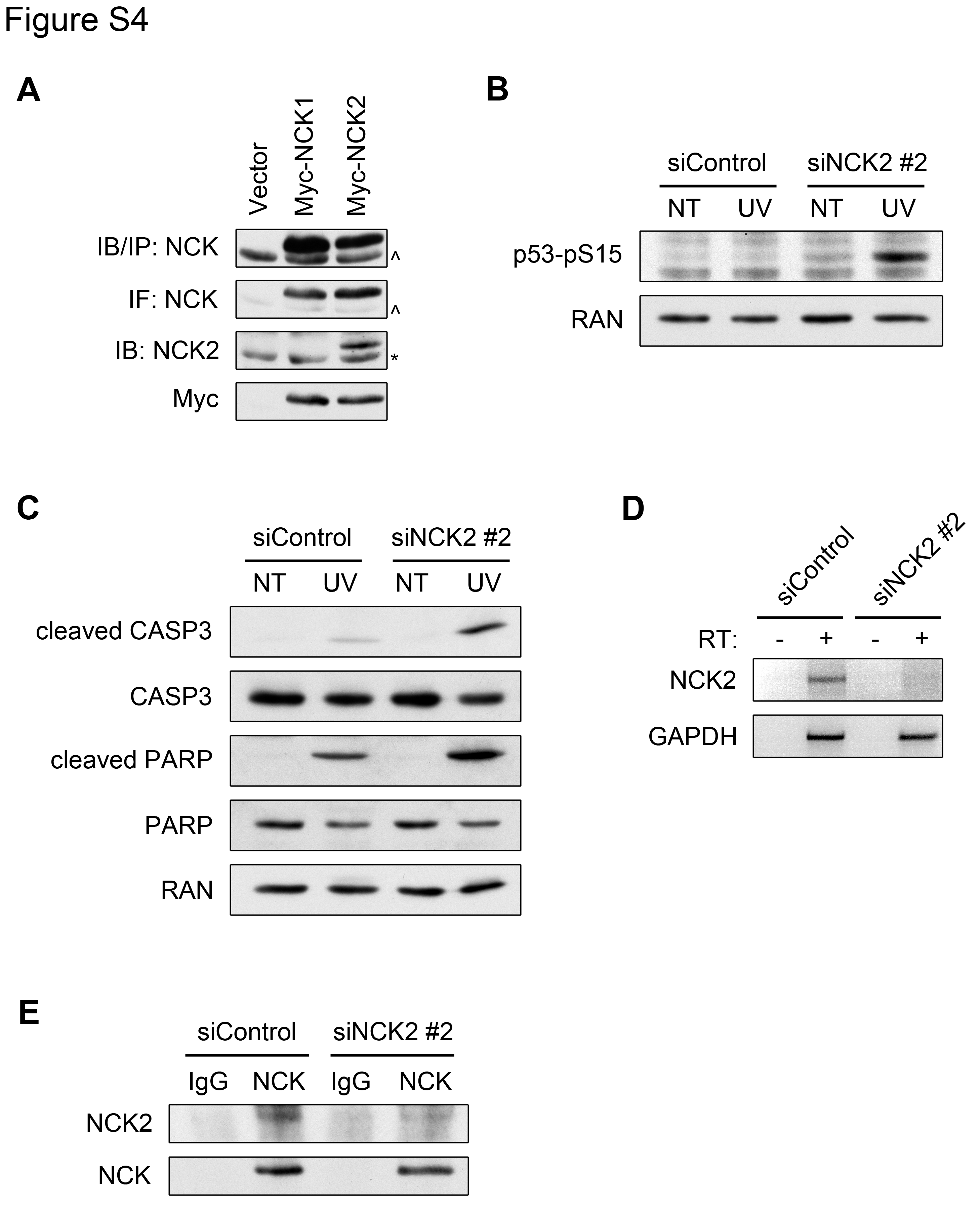

Supplement: Figure S4 — Isoform specificity of NCK antibodies used and experiments with additional NCK2 siRNA. (A) Equal amounts of lysates from 293T cells transfected with myc-vector, myc-NCK1, or myc-NCK2 were immunoblotted with indicated NCK antibodies and Myc. IB indicates antibody was used for immunoblots. IP indicates antibody was used for immunoprecipitations. IF indicates antibody was used for immunofluorescence. ^ indicates endogenous NCK. * indicates non-specific band. n = 3. (B) HeLa cells transfected with control, or NCK2 siRNA#2 were treated with 50 J/m2 UV and allowed to recover for 2 hr before lysates were prepared. Equal amounts of lysates were immunoblotted for p53-pS15 (phospho-specific). RAN was used as a loading control. n = 3. (C) Cells were treated the same as in B, and equal amounts of lysates were immunoblotted for cleaved CASP3, total CASP3, cleaved PARP, and total PARP. RAN was used as a loading control. n = 3. (D) RT-PCR of NCK2 mRNA from HeLa cells transfected with control, or NCK2 siRNA#2. GAPDH was used as an internal control. RT = reverse transcriptase. n = 3. (E) Cell lysates from HeLa cells transfected with control, or NCK2 siRNA#2 were immunoprecipitated with a NCK antibody, which detects NCK 1 and NCK2, or IgG control, and immunoblotted for NCK or NCK2. n = 3. (TIF) [file pone.0076204.s004.tif]
